# Resting and exercise metabolic characteristics in obese children with insulin resistance

**DOI:** 10.3389/fphys.2022.1049560

**Published:** 2022-12-02

**Authors:** Cao Youxiang, Zhu Lin, Chen Zekai, Xie Weijun

**Affiliations:** ^1^ Graduate Department of Guangzhou Sport University, Guangzhou Sport University, Guangzhou, China; ^2^ School of Sport and Health, Guangzhou Sport University, Guangzhou, China

**Keywords:** insulin resistance, obesity, children/adolescents, FATmax, fat metabolic flexibility

## Abstract

**Purpose:** This study aimed to explore the characteristics of resting energy expenditure (REE) and lipid metabolism during incremental load exercise in obese children and adolescents with insulin resistance (IR) to provide evidence for exercise intervention in obese children and adolescents with IR.

**Method:** From July 2019 to August 2021, 195 obese children and adolescents aged 13–17 were recruited through a summer camp. The participants were divided into IR (*n* = 67) and no-IR (without insulin resistance, *n* = 128) groups and underwent morphology, blood indicators, body composition, and resting energy consumption gas metabolism tests. Thirty participants each were randomly selected from the IR and no-IR groups to carry out the incremental treadmill test.

**Results:** Significant metabolic differences in resting and exercise duration were found between the IR and no-IR groups. In the resting state, the resting metabolic equivalents (4.33 ± 0.94 ml/min/kg vs. 3.91 ± 0.73 ml/min/kg, *p* = 0.001) and REE (2464.03 ± 462.29 kcal/d vs. 2143.88 ± 380.07 kcal/d, *p* < 0.001) in the IR group were significantly higher than in the no-IR group. During exercise, the absolute maximal fat oxidation (0.33 ± 0.07 g/min vs. 0.36 ± 0.09 g/min, *p* = 0.002) in the IR group was significantly lower than in the no-IR group; maximal fat oxidation intensity (130.9 ± 8.9 bpm vs. 139.9 ± 7.4 bpm, *p* = 0.040) was significantly lower in the IR group.

**Conclusion:** Significant resting and exercise metabolic differences were found between obese IR and no-IR children and adolescents. Obese IR children and adolescents have higher REE and lower maximal fat oxidation intensity than obese no-IR children and adolescents.

## Introduction

The prevalence of obesity in children and adolescents has become a global public health problem (Collaborators et al., 2017), and the incidence of obesity-related metabolic diseases, such as cardiovascular disease, type 2 diabetes mellitus, and insulin resistance (IR), has increased annually (Gastaldelli et al., 2007; Neeland et al., 2012; Neeland et al., 2013). Studies revealed that the prevalence of IR among obese children and adolescents was 44.3%, which is much higher than the prevalence in overweight (28.6%) and normal weight (8.9%) children and adolescents (Yin et al., 2013). In the past studies, IR refers to an abnormal state of decreased insulin sensitivity in body tissues, such as the liver or muscle. It was not only the pathophysiological basis of many metabolic diseases (Reaven, 1988; Lee, 2006), but also an important risk factor for cardiovascular diseases (Adeva-Andany et al., 2019). IR in children and adolescents also leads to an increased incidence of cardiovascular disease in adulthood (Yajnik et al., 2015). IR was usually accompanied by beta-cell decompensation, which was more rapid in children and adolescents than in adults and more likely to result in complications (Copeland et al., 2011).

Researchers have suggested that the high incidence of obesity-induced IR may be closely related to abnormalities in glucolipid metabolism, lipid accumulation, and mitochondrial dysfunction (Befroy et al., 2007; Boudina and Graham, 2014; Girousse et al., 2018). As a metabolic disease induced by obesity, IR causes population undergone skeletal muscle mitochondrial dysfunction with reduced ATP synthesis capacity and impaired lipid oxidation (Stump et al., 2003), (i.e., impaired energy substrate conversion), which is central to the pathophysiology associated with IR (Galgani et al., 2008). Current studies on functional substrate related research in IR populations were mostly based on positive energy balance (i.e., energy intake exceeds energy expenditure, like oral glucose tolerance test or high-fat diet intervention), while there were fewer studies related to functional characteristics of substrates in negative energy balance (i.e., energy expenditure exceeds energy intake, through exercise, for instance).

Exercise can be effective in improving IR (Marson et al., 2016; Lee et al., 2019), and exercise intensity is an important factor in the effectiveness of interventions. As far as we know, there are few studies related to resting energy expenditure (REE) in obese IR children and adolescents, which has led to the lack of corresponding diagnostic criteria for exercise intensity in this population. REE is an essential component of daily energy needs and accounts for approximately 60%–70% of total energy expenditure in resting state (Johnstone et al., 2005), and recognized as deriving from biochemical reactions at subcellular and cellular levels. During exercise, as exercise intensity increases, the rate of fat oxidation reaches a maximum at a certain intensity (Brooks and Mercier, 1985), at which time the exercise intensity reaches maximal fat oxidation intensity (FATmax). Theoretically the maximum amount of fat can be consumed when exercising at FATmax (Jiang et al., 2020). Fat accumulation was a major causative factor for IR, and exercise intervention with FATmax to improve IR is well worth exploring. However, few studies have been conducted on the exercise lipid metabolic characteristics of obese children and adolescents with IR, so investigating resting and exercise metabolic characteristics can provide a theoretical reference for IR treatment.

We hypothesized that obese IR children and adolescents have higher REE and lower FATmax than obese no-IR children and adolescents. In this study, we investigated the basal metabolic levels of obese IR children and adolescents. We also investigated the FATmax levels of obese IR children and adolescents during the incremental treadmill test and the lipid metabolism characteristics at different exercise intensities to provide theoretical guidance for exercise intervention for this population.

## Participants and methods

### Participants

This study was conducted with obese children and adolescents who participated in a summer camp in South China (Huizhou, China) between July 2019 and August 2021. The screening criteria were as follows (Collaborators et al., 2017): age between 13 and 17 years (Neeland et al., 2012); the diagnosis of obesity according to the Chinese standard, which was developed based on Chinese children and adolescents (GoCOT, 2004); and (Neeland et al., 2013) able to cooperate with the researchers to complete relevant tests. The exclusion criteria were as follows (Collaborators et al., 2017): morbidly obese children and adolescents with severe obesity complications (≥40 kg/m^2^; confirmed cardiovascular disease, obstructive sleep apnoea, musculoskeletal problems, or idiopathic intracranial hypertension) (Neeland et al., 2012); unable to complete the exercise test (Neeland et al., 2013); taking medication for obesity or other diseases; and (Gastaldelli et al., 2007) fasting plasm glucose (FPG) > 5.60 mmol/L. All the subjects and their parents were informed of the benefits and possible risks associated with the experiment and signed an informed consent form before the experiment. The subjects finished the self-reported pubertal development scale, which can effectively evaluate the puberty development stage of Chinese children and adolescents (Zhu and Chen, 2012). This study was approved by the Ethics Committee of the Guangzhou Sports Institute (Approval number: 2018LCLL-008).

**FIGURE 1:**
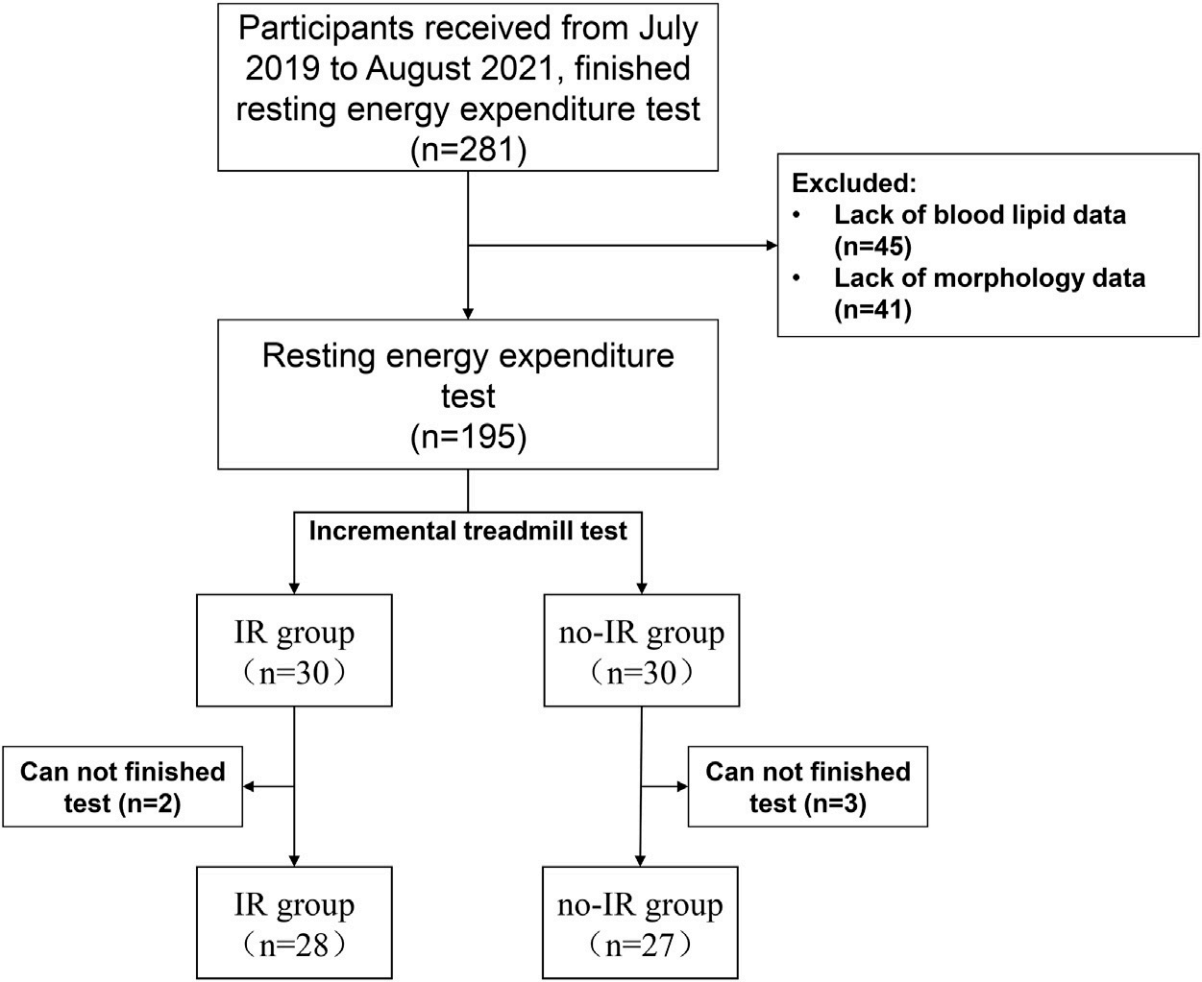
Participants screened flow.

A total of 195 eligible subjects were enrolled in this study, underwent basic information collection, and completed the resting energy test. Thirty participants each were randomly selected from the IR and no-IR groups for the exercise energy test. A tatal of 28 participants in the IR group and 27 in the no-IR group finished the test (Figure 1).

### Morphology and blood indices

Height, body weight (BW), waist circumference (WC), hip circumference (HC), and body composition were measured in the morning after fasting overnight. Height was measured to the nearest 0.1 cm using a standard height meter, and the BW was measured to the nearest 0.1 kg on a digital scale. Body mass index (BMI) was calculated by weight in kilograms divided by the square height in meters. WC and HC were measured using an inelastic plastic fibre tape measure placed directly on the skin at the midpoint between the lower border of the rib cage and the iliac crest (WC) (Autonomous, 1995) and at the maximum extension of the buttocks (HC) (Wang et al., 2000). The participants wore short sleeves and shorts and were not allowed to wear any metal accessories. Body composition was measured using a body composition meter (Inbody, 370, Korea). The subjects kept quiet until the end of the test.

Fasting venous blood samples were collected in the early morning, and the upper layer of the plasma was extracted after low-temperature centrifugation and stored in a refrigerator at −80°C for testing. FPG was measured using the glucose oxidase method, and fasting insulin (FINs) was measured through the electrochemiluminescence method, and total cholesterol (TC), triglycerides (TG), low-density lipoprotein cholesterol (LDL-c), and high-density lipoprotein cholesterol (HDL-c) were tested and analysed using a fully automated biochemical analyser (AU5800, Beckman, Japan). The homeostasis model assessment index of insulin resistance (HOMA-IR) was calculated according to Formula (Collaborators et al., 2017) for the differential diagnosis of IR, and the criterion was HOMA-IR > 3 (Yin et al., 2013).

$$HOMA-IR=\frac{FINs\left(\frac{\mu U}{L}\right)*FPG\left(\frac{mmol}{L}\right)}{22.5}$$ (1)

### Indirect calorimetry measurement

REE and incremental load exercise energy consumption were measured using indirect calorimetry, which is the gold standard for energy metabolism measurements (Boullata et al., 2007). Energy consumption tests were performed using a portable gas metabolic meter (Cortex Meta Max 3B, CORTEX, Germany). After warming up the instrument, the ambient gas (before each test), barometric pressure (once a day), and volume (once a day) were set, and standard gas calibration was performed once a month. The standard gases are 15% O_2_, 5% CO_2_, and 80% N_2_ (CORTEX, Germany).

The REE test was performed in the supine position in the morning under a fasted state. All the participants were kept awake and quiet during the test, and physical activities were prohibited. The room temperature was 25–27°C, and the participants’ carbon dioxide production (VCO_2_) and oxygen consumption (VO_2_) were recorded continuously for 15 min.

The FATmax test was performed using an incremental load treadmill test. Given that the recommended minimum phase duration to reach a steady metabolic state for obese children and adolescents is no less than 3 min (Bordenave et al., 2007; Brun et al., 2011; Chrzanowski-Smith et al., 2018), the exercise test time for each phase was 5 min. The initial speed of the incremental load was 3 km/h, followed by 1 km/h stepwise increments, to 4 km/h, 5 km/h, 6 km/h, and 7 km/h, respectively. The termination criterion was respiratory exchange rate (RER) > 1.0 (Macfarlane, 2017), or exhaustion. During the test, the participants wore a heart rate monitor (H7, Polar, Finland) to monitor their heart rate (HR) in real time.

### Calculation

The first 1–10 min was the adaptation period, and 11–15 min was the recorded average to calculate VCO_2_ and VO_2_. The adaptation period was 1–3 min for each level of the incremental treadmill test, and the average VCO_2_ and VO_2_ at each level were calculated by intercepting 4–5 min of data. The fat oxidation levels were calculated according to Frayn (Eqs 2, 3) (Frayn, 1983). For each subject, the calculated values of fat oxidation were depicted graphically as a function of exercise intensity, and a third polynomial fitting curve with the intersection in (0,0) was constructed to determine the relative exercise intensities that elicited FATmax and the maximal fat oxidation rate (FOmax, g/min) (Achten et al., 2002). Metabolic flexibility was defined as the difference between the FOmax and the resting fat oxidation. REE was calculated using the Weir formula (Eq. 4) (Weir, 1949), and the RER was calculated from VCO_2_ and VO_2_ (Eq. 5).

FO=1.67×VO2−1.67×VCO2 (2)

CHO= 4.55×VCO2−3.21×VO2 (3)

$$REE=3.941\times VO2\left(L/\min\right)+1.11\times VCO2\left(L/\min\right)$$ (4)

$$RER=\frac{VCO2\left(L/\min\right)}{VO2\left(L/\min\right)}$$ (5)

### Statistical analysis

GraphPad Prism 8 (GraphPad Prism Software Inc., San Diego, CA) was used to draw violin plots and line graphs. Statistical package for social sciences (SPSS, version 24, IBM Corporation, United States) software was used for the statistical analysis of the data. Continuous variables were tested for normality using the Kolmogorov–Smirnov method, and the data conforming to a normal distribution are expressed as mean ± standard deviation (mean ± SD). One-way ANOVA was used to analyze the differences between groups. The data that did not follow a normal distribution were expressed as median (quartiles) [M (P25, P75)], and the Mann–Whitney U test was used. Pearson correlation was used to determine the relationships between REE and fat free mass (FFM). For all the statistical analyses, significance was accepted at *p* < 0.05. Categorical variables were compared between group differences using chi-square tests. The relevant influence factors of REE were explored by multiple linear regression.

## Results

### Participants characteristics

A total of 195 eligible participants were included in this study and divided into the IR group (*n* = 67) and the no-IR group (*n* = 128); the prevalence of IR was 34.36%. BW, WC, HC, body fat (BF), body fat percentage (BFP), TC, and TG were significantly higher in the IR group than in the no-IR group (*p* < 0.05), whereas there was no significant difference between the FFM, FPG, LDL-c, and HDL-c of the IR and no-IR groups (*p* > 0.05). Among the IR group, 90.3% were in late adolescence or post-puberty, whereas 92.1% of the no-IR group were in this stage (Table 1).

**TABLE 1:** Baseline characteristics of the participants.

	Total (*n* = 195)	IR Group (*n* = 67)	No-IR Group (*n* = 128)	*p*-value
Age	14.01 ± 1.00	14.12 ± 1.07	13.95 ± 0.97	0.312
Male/Female	102/93	38/29	64/64	0.373
Height (cm)	165.37 ± 7.94	166.86 ± 7.58	164.59 ± 8.05	0.057
BW (kg)	82.13 ± 12.35	84.94 ± 12.74	80.67 ± 11.94	0.022
BMI	29.99 ± 3.55	30.49 ± 3.47	29.73 ± 3.58	0.160
WC (cm)	99.69 ± 10.05	101.67 ± 10.24	98.66 ± 9.84	0.047
HC (cm)	106.90 ± 7.75	109.04 ± 7.78	105.79 ± 7.52	0.005
BFP	33.26 ± 5.89	34.57 ± 6.68	32.58 ± 5.33	0.024
FFM (kg)	53.71 ± 8.64	54.90 ± 9.60	53.07 ± 8.09	0.161
BF (kg)	28.40 ± 8.17	30.03 ± 8.58	27.60 ± 7.79	0.047
FINs (μU/L)	12.87 ± 10.32	21.98 ± 12.48	8.10 ± 3.88	< 0.001
FPG (mmol/L)	4.91 ± 0.96	4.97 ± 0.85	4.88 ± 1.02	0.569
TC (mmol/L)	4.26 ± 1.33	4.58 ± 1.80	4.09 ± 0.93	0.014
HDL-c (mmol/L)	1.13 ± 0.23	1.11 ± 0.21	1.14 ± 0.23	0.389
TG (mmol/L)	1.02 ± 0.54	1.19 ± 0.58	0.93 ± 0.50	0.002
LDL-c (mmol/L)	2.38 ± 0.74	2.48 ± 0.75	2.33 ± 0.73	0.192
HOMA-IR	2.76 ± 2.37	4.80 ± 3.01	1.69 ± 0.71	< 0.001
Stages of puberty	143	41	102	
Mid-Pubertal	12 (8.4%)	4 (9.7%)	8 (7.9%)	
Late Puberty	28 (19.6)	11 (26.8%)	17 (16.7%)	
Postpubertal	103 (72.0%)	26 (63.4%)	77 (75.5%)	

BMI, body mass index; BF, body fat; BFP, body fat percent; BW, body weight; FFM, Fat Free Mass; FPG, fasting plasma glucose; FINs, fasting insulin; HOMA-IR, Homeostasis Model Assessment index of Insulin Resistance; HC, hip circumference; HDL-c, high-density lipoprotein cholesterol; LDL-c, low-density lipoprotein cholesterol; TC, Total cholesterol; TG, Triglycerides; WC, waist circumference.

### Resting metabolic test results

The resting metabolism test results showed a significant difference between the IR and no-IR groups. The metabolic equivalent (MET) (4.33 ml/min/kg vs. 3.91 ± 0.73 ml/min/kg, *p* = 0.001) and REE (2464.03 ± 462.29 kcal/d vs. 2143.88 ± 380.07 kcal/d, *p* < 0.001) were significantly higher in the IR group than in the no-IR group. The proportion of lipid energy supply at rest was significantly lower in the IR group than in the no-IR group (0.55 ± 0.20 vs. 0.62 ± 0.20, *p* = 0.033). REE was highly correlated with FFM in the IR group (r = 0.803, *p* < 0.001) and no-IR group (r = 0.592, *p* < 0.001), and the correction between FFM and REE was higher in the IR group than in the no-IR group. When REE was normalized for FFM, REE/FFM was found to be significantly higher in the IR group than in the no-IR group (*p* = 0.002). BF and BFP were significantly higher in the IR group than in the no-IR group (*p* < 0.05); after normalized for BF, the results show no significant difference between REE/BF of the IR and no-IR groups (*p* = 0.076) (Table 2).

**TABLE 2:** Characteristics of resting metabolic in IR and no-IR group.

	Total (*n* = 195)	IR group (*n* = 67)	No-IR group (*n* = 128)	*p*-value
VO2 (ml/min)	330.54 ± 63.16	359.58 ± 66.93	315.35 ± 55.58	< 0.001
VCO2 (ml/min)	250.98 ± 58.18	280.71 ± 61.18	235.42 ± 50.16	< 0.001
1MET (ml/min/kg)	4.06 ± 0.83	4.33 ± 0.94	3.91 ± 0.73	0.001
RER	0.77 ± 0.05	0.78 ± 0.04	0.77 ± 0.04	0.021
REE (kcal/d)	2253.88 ± 436.47	2464.03 ± 462.29	2143.88 ± 380.07	< 0.001
REE/BW (kcal/d/kg)	27.60 ± 4.51	29.17 ± 4.62	26.77 ± 4.24	< 0.001
REE/FFM (kcal/d/kg)	42.50 ± 9.98	46.59 ± 12.76	40.95 ± 8.08	0.002
REE/BF (kcal/d/kg)	84.95 ± 27.72	89.81 ± 34.72	82.41 ± 22.99	0.076
FO (g/min)	0.12 ± 0.03	0.13 ± 0.04	0.12 ± 0.03	0.024
CHO (g/min)	0.10 ± 0.07	0.12 ± 0.07	0.09 ± 0.07	0.002
Proportion of FO	0.60 ± 0.20	0.55 ± 0.20	0.62 ± 0.20	0.033

BF, Body Fat; BW, Body Weight; CHO, Carbohydrate Oxidation; FO, Fat Oxidation; FFM, Fat Free Mass; MET, Metabolic Equivalent; Ratio of FO, Proportion of fat oxidation; REE, Resting Energy Expenditure; RER, Respiratory Exchange Rate; VCO_2_, Carbon Dioxide Production; VO_2_, Oxygen Consumption.

### Multiple linear regression analysis of resting energy expenditure

Multiple linear regression analysis was performed using REE as the dependent variable and gender, age, pubertal grade, FFM, BF, and HOMA-IR as independent variables (Table 3). The results revealed that REE was significantly correlated with FFM and HOMA-IR levels (*p* < 0.05), but not with gender, age, pubertal stage, and BF (*p* > 0.05).

**TABLE 3:** Multiple linear regression analysis of resting energy expenditure.

Independent variable	β-coefficient	*p*-value	Model *R* ^2^	Model *p*-value
Gender	126.894	0.185	0.249	< 0.001
Age	18.522	0.513		
Puberty	−90.181	0.084		
FFM	11.198	0.047		
BF	−1.041	0.841		
HOMA-IR	55.452	< 0.001		

BF, Body Fat; FFM, Fat Free Mass; HOMA-IR, Homeostasis Model Assessment index of Insulin Resistance.

### Heart rate response to the incremental treadmill test

**FIGURE 2:**
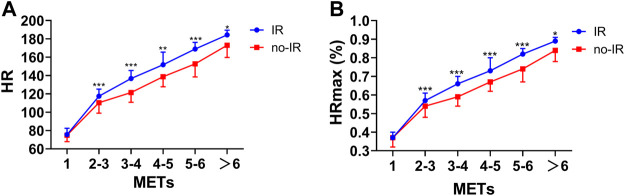
HR and HRmax (%) response to the incremental treadmill test in the IR group and no-IR group. Line graph for theComparsion of HR response in different METs. **(A)**: Comparsion of HR response to incremental treadmill test in the IR group and no-IR group; **(B)**: Comparsion of HRmax (%) response to incremental treadmill test in the IR group and no-IR group. *p < 0.05, **p < 0.01, ***p < 0.001 between IR and no-IR groups.

During the test, HR gradually increased with intensity, and HR response was significantly higher in the IR group than in the no-IR group. Under the same intensity, HR and HRmax% were significantly higher in the IR group than in the no-IR group (*p* < 0.05) (Figure 2).

### Fat and carbohydrate metabolic response to the incremental treadmill test

**FIGURE 3:**
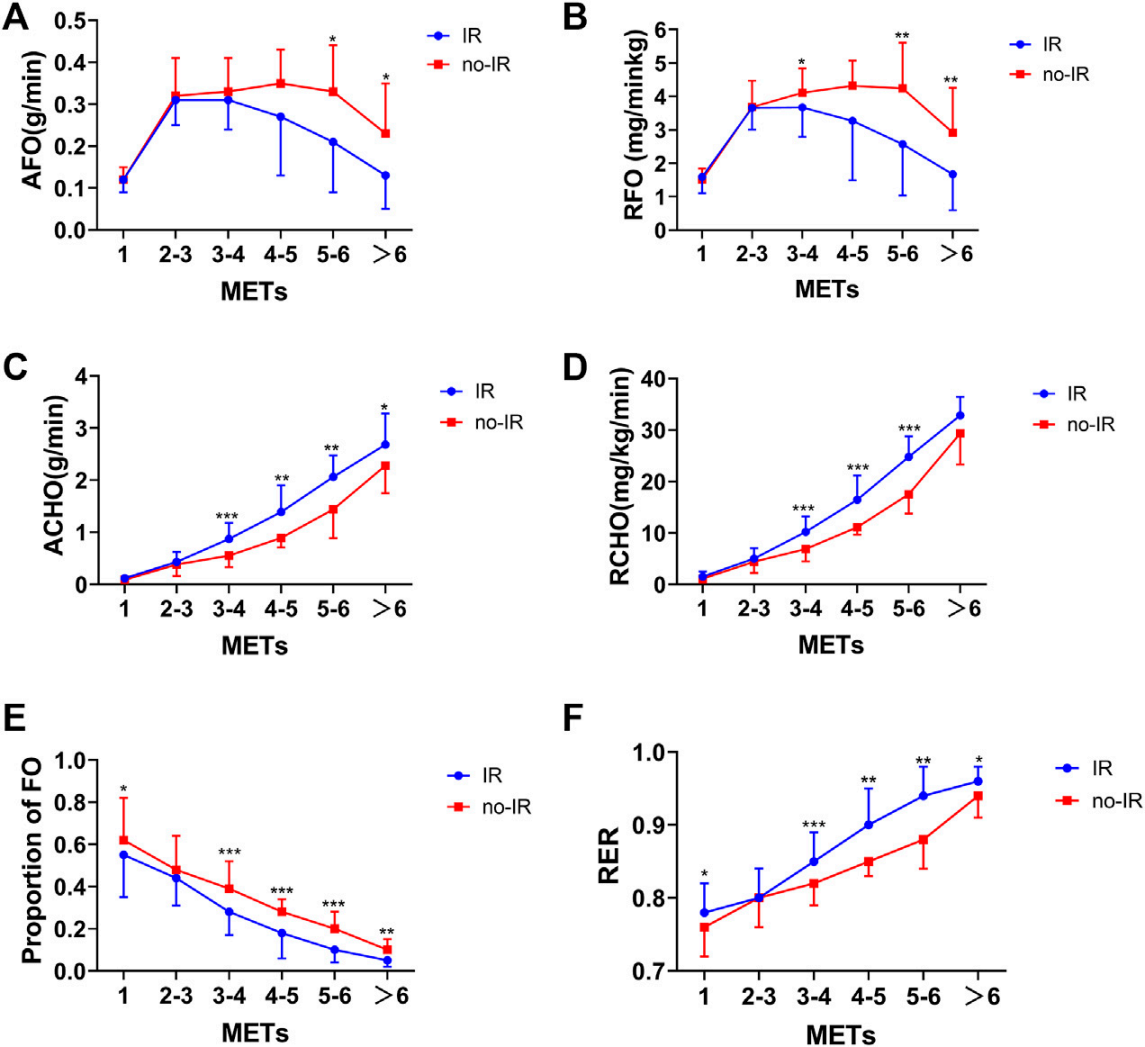
Fat and carbohydrate oxidation response to incremental treadmill test in IR and no-IR groups. Line graph of metabolic characteristics at different intensities. **(A)**: AFO response to incremental treadmill test in IR and no-IR groups; **(B)**: RFO response to incremental treadmill test in IR and no-IR groups; **(C)**: Absolute carbohydrate oxidation (ACHO) response to incremental treadmill test in IR and no-IR groups; **(D)**: Relative carbohydrate oxidation (RCHO) response to incremental treadmill test in IR and no-IR groups; **(E)**: Proportion of FO during incremental treadmill test; **(F)**: RER changes during incremental treadmill test in IR and no-IR groups. *P < 0.05, **P < 0.01, ***P < 0.001 between IR and no-IR groups.

The incremental treadmill test revealed that the absolute fat oxidation (AFO) increased as with the intensity in both the IR and no-IR groups. The absolute fat oxidation of the IR and no-IR groups decreased with increasing intensity when the exercise intensity exceeded 4 and 5 METs, respectively. Further analysis of the relative fat oxidation (RFO) of the IR and no-IR groups revealed that the RFO of the IR group was significantly lower than that of the no-IR group when the exercise intensity exceeded 3 METs (*p* < 0.05). In addition, the absolute carbohydrate oxidation energy supply level and the relative carbohydrate oxidation were also significantly higher in the IR group than in the no-IR group when the exercise intensity exceeded 3 METs (*p* < 0.05 and *p* < 0.001, respectively). When the exercise intensity exceeded 3 METs, the proportion of lipid energy supply was significantly lower in the IR group than in the no-IR group at the same exercise intensity (*p* < 0.01). The carbohydrate energy supply in the IR group was significantly higher than in the no-IR group when the exercise intensity exceeded 3 METs. The proportion of fat energy supply was lower in the IR group than in the no-IR group when the exercise intensity exceeded 3 METs during exercise (Figure 3).

### Fat oxidation and FATmax in insulin resistance and no-insulin resistance groups

**FIGURE 4:**
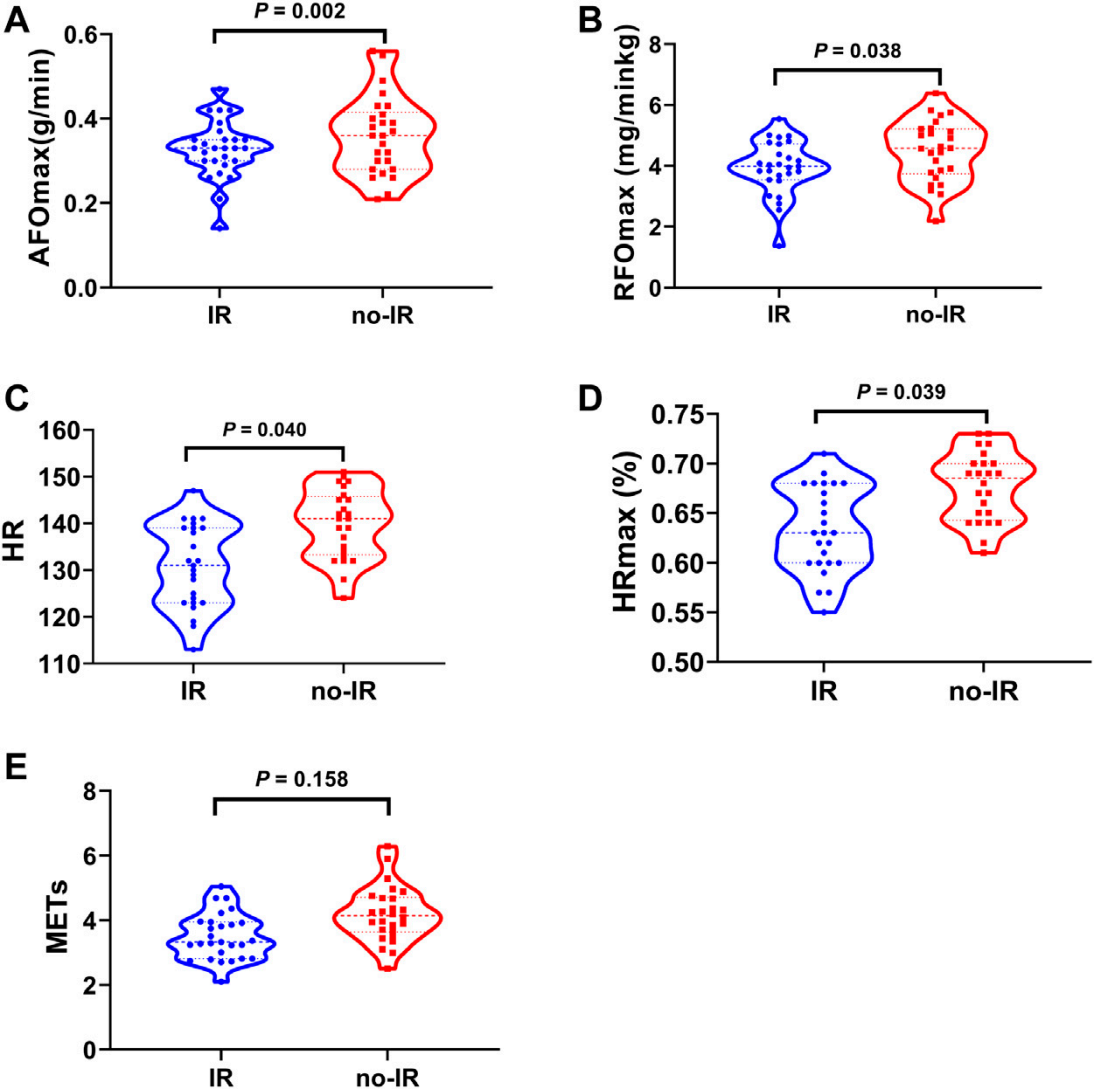
Fat Oxidation and FATmax in IR and no-IR groups. Violin plot of fat oxidation characteristics between IR and no-IR group. **(A)**: Absolute maximal fat oxidation rate (AFOmax) between IR and no-IR groups; **(B)**: Relative maximal fat oxidation rate (RFOmax) between IR and no-IR groups; **(C)**: FATmax (HR) between IR and no-IR groups; **(D)**: FATmax (HRmax %) between IR and no-IR groups; **(E)**: FATmax (METs) between IR and no-IR groups.

Calculations attained by fitting a third order polynomial fit curve for fat oxidation kinetics for each participant revealed that the absolute maximal fat oxidation was significantly lower in the IR group than in the no-IR group (0.33 ± 0.07 g/min vs. 0.36 ± 0.09 g/min, *p* = 0.002) The relative maximal fat oxidation was also lower in the IR group than in the no-IR group (3.93 ± 0.89 mg/min/kg vs. 4.49 ± 1.00 mg/min/kg, *p* = 0.038). When the exercise intensity reached FATmax, the IR group had significantly lower HR values (130.9 ± 8.9 bpm vs. 139.9 ± 7.4 bpm, *p* = 0.040) and a lower HRmax% than the no-IR group (0.63 ± 0.04 vs. 0.68 ± 0.03, *p* = 0.039); the METs of the IR and no-IR groups showed no significant difference (3.50 ± 0.71 vs. 4.19 ± 0.86, *p* = 0.158) (Figure 4).

### Fat metabolic flexibility in insulin resistance and no-insulin resistance groups

**FIGURE 5:**
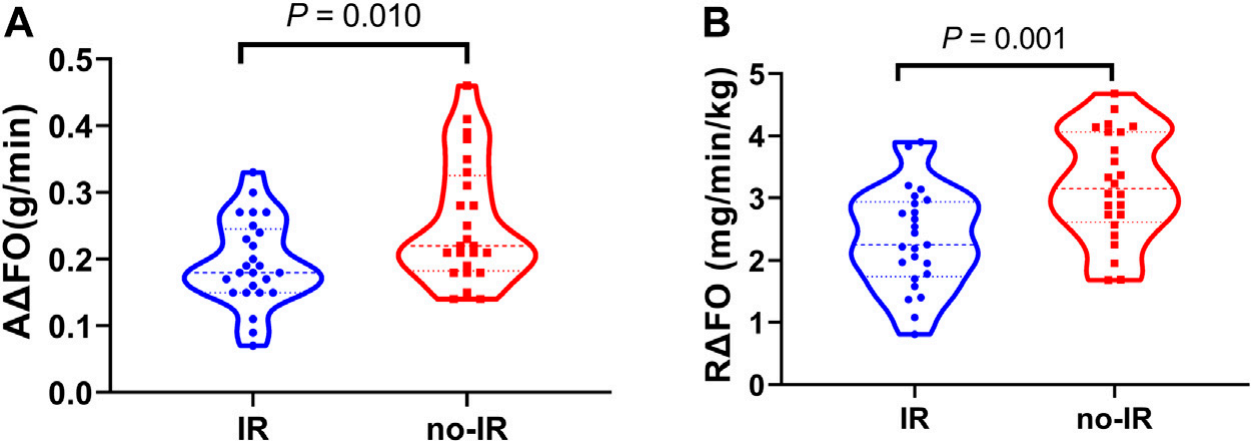
Fat metabolic flexibility between IR and no-IR groups. Violin plot of fat metabolic flexibility between IR and no-IR group. **(A)**: Absolute fat metabolic flexibility (AΔFO) between IR and no-IR groups; **(B)**: Relative fat metabolic flexibility (RΔFO) between IR and no-IR groups.

Significant differences in fat metabolic flexibility were observed during the incremental treadmill test. Absolute fat metabolic flexibility was significantly lower in the IR group than in the no-IR group (0.19 ± 0.06 g/min vs. 0.25 ± 0.09 g/min, *p* = 0.010); after adjusting the body weight factors, relative fat metabolic flexibility remained significantly lower in the IR group than in the no-IR group (2.34 ± 0.80 mg/min/kg vs. 3.20 ± 0.87 mg/min/kg, *p* = 0.001) (Figure 5).

## Discussion

The main findings of this study are as follows (Collaborators et al., 2017): The energy metabolism characteristics of the IR and no-IR groups have a significant difference. The 1 MET and REE in the IR group were significantly higher than in the no-IR group, and the proportion of fat supply was significantly lower in the IR group (Neeland et al., 2012). The absolute maximal fat oxidation rate was significantly lower in the IR group, and the FATmax was also smaller in the IR group (Neeland et al., 2013). During the incremental treadmill test, the rate of fat oxidation in the no-IR group was significantly higher than in the IR group at the same exercise intensity, whereas the HR response was milder (Gastaldelli et al., 2007). The fat metabolic flexibility was significantly lower, and the level of fat mobilization was weaker in the IR group than in the no-IR group.

The diagnosis of obesity according to the Chinese standard. Because the participants were Chinese, the obesity identification criteria were especially developed for Chinese, which were also in consistent with the eastern Asia ethnic characteristics (GoCOT, 2004). Two international references are widely used: the International Obesity Task Force reference (Cole et al., 2000) and the World Health Organization standard 2007 (de Onis et al., 2007), but the two references applied to the same children lead to different obesity prevalence rates (Wang and Wang, 2002).

The REE in the IR group was significantly higher than in the no-IR group, which was generally consistent with previous studies (Ten et al., 2008). Some studies found that the main influence factors of REE were BW, FFM and metabolic status (Bandini et al., 1990; Goran and Treuth, 2001; Ten et al., 2008). After conducting multiple linear analyses, we found that FFM and HOMA-IR levels were important determinants of REE. Although, the FFM in the IR and no-IR groups did not differ significantly in this study (54.90 ± 9.60 kg vs. 53.07 ± 8.09 kg, *p* = 0.161). The BF in the IR group was significantly higher than in the no-IR group (*p* < 0.05), the main reason may be that FFM (50% of skeletal muscle) has a greater impact on REE than BF (Molnár and Schutz, 1997). FFM consists of highly metabolically active muscle and organs, such as the skeletal muscle’s metabolic rate was 13 kcal/kg/d, and thus the average 25 kg organ was predicted to consume 325 kcal/d or roughly 13% of REE (Heymsfield et al., 2021). Another important factor affecting REE was HOMA-IR. The degree of IR showed a significant positive correlation with REE. This may be a consequence of an increased neoglucogenetic activity. Neoglucogenesis is, an energy costly metabolic pathway responsible for increased FPG (Buscemi et al., 2014). Research also found that the gluconeogenic energy consumption in the IR group accounted for approximately one-third of the total resting energy consumption, which is much higher than in the normal weight healthy group (Quaye et al., 2021).

Reduced glycogen uptake and abnormal lipid metabolism were important basic features of IR (Cline et al., 1999; Kelley et al., 2002). According to the Randle cycle theory, using one energy substance leads to the inhibition of other energy substances. The selection of glucose and fatty acids by the body is a dynamic process (Randle et al., 1963), elevated fatty acid levels lead to glucose intolerance, which induces IR, increases lipolytic reactions, reduces mitochondrial fatty acid oxidation in IR populations, leaded to the accumulation of diacylglycerols and ceramides by reverse synthesis, further stimulating protein kinases and inhibiting insulin signaling (Morino et al., 2006; Savage et al., 2007), created a vicious cycle. The IR population had an 80% higher intracellular triglyceride content in myocytes and a 30% lower rate of ATP synthesis in mitochondria than the insulin-sensitive normal population (DiMenna and Arad, 2018). It was further found that under insulin-stimulated conditions (hyperinsulin-orthoglycemic clamp), the rate of mitochondrial synthesis increased by only 5% in the IR population, compared with a 90% increase in the insulin-sensitive normal population (Petersen et al., 2005). In the present study, fat oxidation was significantly higher in the IR group than in the no-IR group during resting conditions and lower in carbohydrate oxidation, suggesting an increased fatty acid supply and inhibited glycogen oxidation in IR.

Despite higher levels of lipid metabolism in the IR group under resting conditions, the IR group had significantly lower lipid metabolic flexibility than the no-IR group during the incremental treadmill test (0.19 ± 0.06 g/min vs. 0.25 ± 0.09 g/min, *p* = 0.010). Metabolic flexibility refers to the ability of the body to respond to changes in metabolic or energy requirements (Kelley et al., 1999). Normal levels of metabolic flexibility play a critical role in maintaining intracellular energy homeostasis (Goodpaster and Sparks, 2017; Smith et al., 2018). Compared to the normal weight populations, the obese IR population have a weaker oxidative regulation of lipid metabolism *in vivo* before and after high-fat diet intervention (Blaak et al., 2006). Impaired flexibility of lipid metabolism also means that FFA cannot be metabolized efficiently, which may lead to enhanced body lipotoxicity and further aggravate IR.

Exercise intensity is usually based on the gold standard of MET: 1 MET is the rate of energy expenditure at resting state, which is approximately equal to 3.5 ml min^−1^·kg^−1^ (Balke, 1960); 1.51–2.99 METs are for low intensity; 3.00–5.99 METs are for moderate intensity; and ≥6 METs are for high intensity (Hibbing et al., 2020). The 1 MET of the obese IR children and adolescents in this study was significantly higher than that of the no-IR group (4.33 ± 0.94 ml min^−1^·kg^−1^ vs. 3.91 ± 0.73 ml min^−1^·kg^−1^), and the resting metabolic equivalent was also higher than the agreed value of 3.5 ml min^−1^·kg^−1^ in both the IR and no-IR groups. Given that METs have strong standardized properties and generalizability (Hibbing et al., 2020), a redefinition is needed when using MET for exercise intensity evaluation in IR or no-IR obese children and adolescents.

In the incremental treadmill test, the MFO of the IR group was significantly smaller than that of the no-IR group, which is consistent with previous studies (Cancino Ramírez et al., 2018); under the same exercise intensity, both the AFO and RFO of the IR group were lower than those of the no-IR group, and the difference increased in significance with the exercise intensity. This phenomenon may be related to the mitochondrial dysfunction in the adipose tissue of the IR group. The IR population exhibited a lower expression of mitochondrial function-related proteins in the subcutaneous adipose tissue than the insulin-sensitive normal population (Xie et al., 2016). The abnormal mitochondrial function in the adipose tissue also inhibits insulin secretion and reduces insulin sensitivity (Luo and Liu, 2016). Hypoxia-inducible factor-1α (HIF1α) is a central regulator of glycolysis during hypoxia (Sato et al., 2019), sirtuin2 (SIRT2) plays an important role in fatty acid oxidation progress (Cha et al., 2017), peroxisome proliferator-activated receptor *γ* coactivator 1α (PGC-1α) can regulates lipid metabolism by upregulating the expression of several genes of the tricarboxylic acid cycle and the mitochondrial fatty acid oxidation pathway (Cheng et al., 2018). IR populations have higher expression levels of HIF1α and lower expression levels of SIRT2 than the normal weight populations. Abnormal expression levels of HIF-α and SIRT2 reduce PGC-1α deacetylation, resulting in decreased adipose tissue β-oxidation levels (Krishnan et al., 2012). In obese individuals, the body’s lipid metabolism capacity is reduced, the body becomes lipotoxic, and insulin signaling becomes impaired, causing the body to become IR (Morino et al., 2006), which leads to the further accumulation of lipids and subsequently increases obesity.

Exercise is an important prevention method and adjunctive treatment of obesity and chronic diseases (Pedersen and Saltin, 2015). It has a significant effect on improving lipid metabolism disorders, increasing insulin sensitivity, and improving IR in obese children and adolescents (Lee et al., 2012; Marson et al., 2016; Lee et al., 2019). One of the essential ways that exercise improves IR is by increasing lipid oxidation (Pedersen and Saltin, 2006). Obesity is an important causative factor of IR, and efficient fat loss through exercise was an effective way of improving IR, while FATmax was the optimal fat loss intensity (Jiang et al., 2020). A cross-sectional study found that more fat was burned when exercising at FATmax (45%VO_2_max) than at high intensities (70% VO_2_max) (Lazzer et al., 2010). The present study also found that the FATmax was significantly lower in the IR group than in the no-IR group (130.9 ± 8.9 vs. 139.9 ± 7.4, *p* = 0.040), which is consistent with previous studies (Suk et al., 2015). This result suggested that lower exercise intensity was required when fat loss exercise interventions were performed in the obese IR population than in the no-IR group. In addition, the HR response during exercise was more significant in the IR group, which could be mainly due to the lower level of cardiorespiratory fitness in the IR group. Cardiorespiratory fitness in children also shows a significant positive correlation with insulin sensitivity, the lower the level of cardiorespiratory fitness, the lower the insulin sensitivity (Slinger et al., 2008; Henderson et al., 2012; Medrano et al., 2020), which also suggested that the lower the insulin sensitivity during exercise, the stronger the cardiovascular response will be.

Different exercise intensities determined the different energy substrates during exercise, and produced different metabolic responses (Fedewa et al., 2014). Low to moderate intensity was the optimal intensity for fat oxidation, while moderate to high intensity was highly significant for carbohydrate consumption (Houmard, 2008). Metabolomic studies have also found that moderate intensity is more beneficial for lipid oxidation than high intensity for the same amount of exercise (Huffman et al., 2014). In the resting state, the energy supply is dominated by lipid oxidation, and the phosphorylase activity in the skeletal muscle increases with the intensity of exercise, leading to an accelerated rate of glycogen breakdown and a gradual increase in the proportion of carbohydrate supply (Romijn et al., 1993). Obesity is an important causative factor of IR in the body, and exercise at FATmax is theoretically the most beneficial for fat elimination (Jiang et al., 2020) and has the most significant effect on IR improvement while controlling other exercise conditions. Tan et al. (Tan et al., 2018) also found that exercise intervention at FATmax for 12 weeks at 3 times per week significantly improves insulin sensitivity.

## Limitations

This study mainly used indirect calorimetry to explores energy metabolism characteristics and glycolipid metabolism during incremental load exercise in obese IR and no-IR children and adolescents but did not include normal weight children and adolescents. In addition, protein oxidation was not calculated in this study, despite the low percentage of protein energy supply. The present study initially explored the flexibility of lipid metabolism in the IR group. Although other methods like ^13^C-based metabolic flux analysis or metabonomics are more advanced, these methods are disadvantage of invasive, extremely labor-intensive, or time-consuming. In addition, in future research, advanced methods can be used to further explore the metabolic flexibility of IR, the mechanisms and potential treatments for exercise to improve IR or type 2 diabetes mellitus.

## Conclusion

Significant differences were observed in the resting and exercise states of obese IR and no-IR children and adolescent. The REE of the IR group was significantly higher than in the no-IR group. In constrast, FATmax was lower in the no-IR group, and the HR response during the incremental treadmill test was stronger. The obese IR children and adolescents showed lipid metabolic inflexibility.

## Data Availability

The raw data supporting the conclusion of this article will be made available by the authors, without undue reservation.
